# Supplementary material for: Active site specificity profiling datasets of matrix metalloproteinases (MMPs) 1, 2, 3, 7, 8, 9, 12, 13 and 14
Source: Data Brief. 2016 Feb 22;7:299–310. doi: 10.1016/j.dib.2016.02.036 (PMC4777984; doi:10.1016/j.dib.2016.02.036)
Supplement: Supplementary file 10 — Supplementary material [file mmc10.zip › WebPICS_hMMP14_G_1%/P2.html]

 

PICS results


|  |  |
| --- | --- |
| **P2\_A**  31 in 213 sites   14.6 %    effects > 10 perc. pnts.  (vice-versa in brackets)  P3\_A: -12.8 (-11.7)   P3prime\_K: 14.5 (18.7)   P3prime\_L: -11.4 (-11.4) |  |
| **P2\_C**  9 in 213 sites   4.2 %    effects > 10 perc. pnts.  (vice-versa in brackets)  P3prime\_S: 15.6 (10.1) |  |
| **P2\_K**  20 in 213 sites   9.4 %    effects > 10 perc. pnts.  (vice-versa in brackets)  P3\_P: 22.3 (16.5)   P1\_A: 29.5 (17.9) |  |
| **P2\_N**  16 in 213 sites   7.5 %    effects > 10 perc. pnts.  (vice-versa in brackets)  P1\_W: 10.6 (42.5)   P2prime\_C: 10.6 (42.5) |  |
| **P2\_Q**  11 in 213 sites   5.2 %    effects > 10 perc. pnts.  (vice-versa in brackets)  P1\_Y: 23.1 (28.1)   P2prime\_L: 31.4 (11.5)   P3prime\_Q: 21.7 (19.8) |  |
| **P2\_R**  12 in 213 sites   5.6 %    effects > 10 perc. pnts.  (vice-versa in brackets)  P1prime\_F: 11.5 (12.6) |  |
| **P2\_S**  20 in 213 sites   9.4 %    effects > 10 perc. pnts.  (vice-versa in brackets)  P3\_V: 17.3 (12.8)   P1\_S: 24.2 (21.0) |  |
| **P2\_Y**  8 in 213 sites   3.8 %    effects > 10 perc. pnts.  (vice-versa in brackets)  P1prime\_F: 19.8 (14.4)   P2prime\_F: 31.4 (19.3) |  |
